# Advancing universal health coverage in the COVID-19 era: an assessment of public health services technical efficiency and applied cost allocation in Cambodia

**DOI:** 10.1186/s13561-021-00354-8

**Published:** 2022-01-29

**Authors:** Robert John Kolesar, Peter Bogetoft, Vanara Chea, Guido Erreygers, Sambo Pheakdey

**Affiliations:** 1 Abt Associates, Room 125 (Level 1), Building B, Phnom Penh Center, Corner Sihanouk (274) & Sothearos (3) Blvd, Sangkat Bassac, Khan Chamkrarmon, Phnom Penh, Cambodia; 2 University of Antwerp, Faculty of Business and Economics, Antwerpen, Belgium; 3 General Secretariat for the National Social Protection Council, Cambodian Ministry of Economy and Finance, Phnom Penh, Cambodia; 4 Centre d’Etudes et Recherche sur le Développement International (CERDI), Université Clermont Auvergne, Clermont-Ferrand, France; 5 Copenhagen Business School, Copenhagen, Denmark

**Keywords:** Health service efficiency, Social health protection, Costing, Cost allocation, Universal health coverage, Cambodia, Health Care Financing, Public Health Insurance, Input Output Models, Policy Making, Frontier Estimation, Developing Countries

## Abstract

**Background:**

Achieving universal health coverage (UHC) is a global priority and a keystone element of the 2030 Sustainable Development Goals. However, COVID-19 is causing serious impacts on tax revenue and many countries are facing constraints to new investment in health. To advance UHC progress, countries can also focus on improving health system technical efficiency to maximize the service outputs given the current health financing levels.

**Methods:**

This study assesses Cambodia’s public health services technical efficiency, unit costs, and utilization rates to quantify the extent to which current health financing can accommodate the expansion of social health protection coverage. This study employs Data Envelopment Analysis (DEA), truncated regression, and pioneers the application of DEA Aumann-Shapley applied cost allocation to the health sector, enabling unit cost estimation for the major social health insurance payment categories.

**Results:**

Overall, for the public health system to be fully efficient output would need to increase by 34 and 73% for hospitals and health centers, respectively. We find public sector service quality, private sector providers, and non-discretionary financing to be statistically significant factors affecting technical efficiency. We estimate there is potential supply-side ‘service space’ to expand population coverage to an additional 4.69 million social health insurance beneficiaries with existing financing if the public health system were fully efficient.

**Conclusions:**

Public health service efficiency in Cambodia can be improved by increasing utilization of cost-effective services. This can be achieved by enrolling more beneficiaries into the social health insurance schemes with current supply-side financing levels. Other factors that can lead to increased efficiency are improving health service quality, regulating private sector providers, focusing on discretionary health financing, and incentivizing a referral system.

## Introduction

Cambodia has committed to advancing universal health coverage (UHC) which requires expanding population coverage under its social health protection schemes. However, the near-term potential for new government investment is unlikely as tax revenues fall due to pandemic related economic disruption. Most government ministries and institutions, exempting the Ministry of Health, are required to reduce their expenditures by at least 50% of the approved national budget figures for the current year [1]. However, austerity exacerbates health inequities in countries with weak social protection policies [2]. And, countries with higher levels of inequity in income, education, and health are the least efficient in relation to health outcomes [3].

This study assesses the technical efficiency of Cambodian public health services to quantify the extent to which current health sector supply-side resources can accommodate social health protection expansion. In addition, we examine explanatory factors associated with technical efficiency; and, estimate the unit cost of service provision for the major social health insurance reimbursement categories to inform the policy discussion on strategic purchasing and demand-side health financing.

Increasing health sector expenditure may not significantly affect health outcomes when efficiency is low [4]. Thus, performance measurement of public services is essential to ensure quality services and value for money [5]. Inefficiencies in the public health sector are well documented [6]. The 2010 World Health Report asserts that all countries can achieve more with the same resources, conservatively estimating that 20–40% of all healthcare expenditure is wasted [7]. A study assessing overall health system technical efficiency in Asia found Cambodia to be among the countries that can improve use of the current resources [8]. Public sector health expenditure is 47% of Cambodia’s total health expenditure [9]; however, only about 20% of people with an illness or injury first seek care in the public sector [10]. A district level efficiency analysis of public health services in five Cambodian provinces found evidence of sub-optimal performance [11]. Another study concluded that increasing health service utilization and quality could improve public health center efficiency [12]. Finally, a recent healthcare costing study found considerable differences in workload which was inversely correlated with total and unit costs within each facility level which suggests that cost-efficiency could be improved by increasing service volume [13].

Improved efficiency is a central tenant of Cambodia’s high-level policy and strategy documents. The Rectangular Strategy for Growth, Employment, Equity and Efficiency calls for “ensuring efficiency and effectiveness of the public institutions and management of all the resources” as a means towards medium and long-term sustainable development [14]. The National Strategic Development Plan aims to increase public sector efficiency for sustainable development and poverty reduction to achieve the Sustainable Development Goals [15]. And, the National Social Protection Policy Framework calls for enhancing the efficiency, equity, transparency and consistency of the social protection system [16]. The pandemic’s disproportionate impacts on poor and vulnerable communities present opportunities for policy makers to tackle deep-rooted system performance issues with long-term implications for health financing and health systems performance [17].

Healthcare service cost information is needed to support evidence-based, effective and efficient health care reforms [18]. Regularly updated costing data for each facility level can be used to strengthen strategic purchasing and cost containment [19]. For example, cost data can be used to determine social health insurance reimbursement rates as the system shifts from supply to demand-side financing. Increasing the social health insurance reimbursement would incentivize increased service volume, particularly for priority and cost-effective services, thereby increasing efficiency [20, 21]. In addition, cost data can inform budget planning and efficient resource allocation. However, costing exercises are resource intensive and can require significant expense and effort [13]. Therefore, they are infrequent and often limited to a few facilities and health services, particularly in low- and middle-income countries [13, 22].

## Background

Cambodia’s largest social health protection scheme, the Health Equity Fund (HEF), was established to provide free access to health care for the poorest. The scheme reimburses public health facilities user-fees normally paid by the patient. Since 2017, approximately 2.6 million household members have been covered under the HEF, representing about 16.1% of the total population. Free benefits under the HEF have been extended to some informal workers and selected populations (about 93,500 enrollees). In addition, the roll-out of the On-demand ID Poor system in 2020 has increased eligibility to approximately 468,000 new beneficiaries. Currently, the National Social Security Fund (NSSF) members are approximately 2,314,000 including 1,884,000 private sector employees and 430,000 civil servants, retirees, and veterans. Total effective social health protection coverage equates to about one-third of the total population.

Cambodia’s high-level strategy and policy documents including the National Strategic Development Plan 2019–2023 call to advance UHC by increasing population coverage of social health protection to 65% by 2023. Expanding population coverage under the social health protection schemes is expected to increase public health service utilization. However, user-fee reimbursements paid to public health facilities are allocated to pay for staff incentives (60%) and quality improvement (~ 40%). Thus, social health protection scheme reimbursements are likely insufficient to cover the increased costs related to personnel, medications, and commodities associated with increased utilization when population coverage is expanded [20, 21].

The global impacts of COVID-19 are unprecedented. The pandemic affects medium-term economic growth, poverty, government revenues, and government spending; even countries with relatively low case counts are facing substantial reductions in national revenue [17, 23]. Forecasts project that near-term per capita government health spending could slow and even decrease, particularly among low and lower-middle income countries [17]. In Cambodia, the pandemic has caused sharp deceleration in most of the country’s main growth engines including tourism, manufacturing export, and construction. In 2019, these sectors accounted for more than 70% of growth. In 2020, the Cambodian economy registered negative growth of − 3.1% [24]. This has serious impacts on tax revenue and consequentially on government budgets in general, and the public health budget in particular [25]. At the same time, unemployment is increasing financial vulnerability to health shocks with out-of-pocket healthcare expenditure comprising an excessive share of income [26]. Decreased economic output can be expected to lead to decreased utilization of health services that require payment. A 1% decrease in GDP per capita is expected to result in a decline in out-of-pocket healthcare expenditure per capita of nearly 1% [27]. Despite the Cambodian government’s emergency cash transfer program to mitigate the effects of the COVID-19 economic shock among the poor, poverty is likely to have increased [1, 28]. For most lower-middle income countries government revenue is not expected to rebound to pre-pandemic levels until 2024–2025 [17].

Cambodia’s public health system consists of a network of 34 national and provincial-municipal level hospitals, 92 Operational District (OD) referral hospitals, 1222 health centers, and 128 health posts. Hospitals are sub-categorized into four levels: national hospitals and complimentary package of activities (CPA) hospitals levels 1–3 [29]. In 2019, Cambodia had a total of 117 CPA1–3 hospitals with a provincial-municipality average of 4.68 (Std dev. = 3.29), range of one (1) to 12, and a median of four (4).

In relation to health centers, there are 1222 nationwide, with the provincial-municipality average of 48.88 (Std dev. = 31.4), range of five (5) to 113 and a median of 43. Health centers provide a minimum package of activities (MPA) and operate health posts which extend services to hard-to-reach areas. The MPA focuses on preventative and basic curative services; each health center serves approximately 10,000–20,000 people. Health Operational Districts (ODs), responsible for health center oversight, are typically comprised of 10–15 health centers covering about 100,000–200,000 people and a district referral hospital. A summary of services provided by facility level is presented in Table 1.

**Table 1:** Summary of health services provided by facility level

	National Hospitals (1)	Hospital CPA-3 (2)	Hospital CPA-2 (3)	Hospital CPA-1 (4)	Health Center (5)
**Services provided**	Higher-level tertiary care and specialized services treatment and management for complex health problems	100–250 beds, provide CPA-2-1 services plus various specialized services including intensive care and blood transfusion, ear, nose and throat, ophthalmology, and orthodontic services	60–100 beds, provide CPA-1 services plus emergency care, major surgery and other specialized services including intensive care and blood transfusion, ear, nose and throat, ophthalmology, and orthodontic services	40–60 beds, provide basic obstetric care, but with no major surgery nor general anesthesia; and no blood bank or blood deposit	Preventive and basic curative and delivery services, supplemented by specific activities for vertical programs

Based on [30, 31]

There is empirical evidence demonstrating that good governance and strengthened public financial management systems can positively impact health system performance and service delivery as well as efficiency of government spending on health [17, 32]. In 2001, the Cambodian central government began introducing decentralization reforms [33] with the Public Financial Management Reform Program following in 2004. This program focuses on four areas to improve: (1) budget credibility; (2) financial accountability; (3) budget policy linkages; and (4) performance accountability [34]. The commitment to these reforms was renewed in the government’s high-level strategy and policy documents. The Rectangular Strategy Phase IV sets out to deepen “reforms to achieve good governance, particularly public administration reform, public financial management reform, decentralization and de-concentration reform” [14]. The National Strategic Development Plan 2019–2023 aims to strengthen the implementation of the Decentralization and De-concentration (D&D) Reform Program by delegating power and transfer of functions, resources and technology to administrative units to obtain appropriate autonomy, including decision-making, management and resource allocation [15]. This focus aligns with the National Social Protection Policy Framework’s cross-cutting principles of good governance and effective spending [16].

Until 2020, the Ministry of Health was solely responsible for the organization and delivery of government health services. The Directorate General for Health oversaw health service delivery through 24 Ministry of Health Provincial Health Departments (PHDs) and the municipality of Phnom Penh. Each PHD operated the provincial hospital and governed the Health Operational Districts [30]. In December 2019, the government issued Sub-Decree No. 193 ANKr. BK to delegate health management functions and service provision to the 25 provincial-municipal administrations. This directive transfers decision-making and responsibility for health service management including financial resources, properties, and human resources to the provincial-municipal administrations as of January 1, 2020. In turn, the administrations are “accountable and responsible to Minister of Health for management, organization, and performance of the delegated health functions” in line with the national health policy, strategic plan, clinical guidelines, protocols, and technical standards [35]. In 2019, 38.6% of the approved Ministry of Health budget (~USD $444 million) was allocated to the provincial-municipal level.

## Methods

This study aims to quantify the extent to which current health financing can accommodate the expansion of social health protection coverage in Cambodia. We used data aggregated at the provincial-municipal administration level for all 24 provinces and the municipality of Phnom Penh; this approach enables the inclusion of all public CPA1–3 hospitals and all public health centers in Cambodia. The study design employs Data Envelopment Analysis, truncated regression analysis, and Aumann-Shapley applied cost allocation to estimate public health services technical efficiency, identify explanatory factors, and compute health service unit costs. These parameters are used to calculate the supply-side ‘service space’ potential to expand population coverage to additional social health insurance beneficiaries with existing financing if the public health system were fully efficient. Data sources and analytical methods are described below.

Data was compiled from multiple sources including: the Cambodia Demographic and Health Survey, 2019 Cambodian Census, 2019–2020 Cambodia Socio-economic Survey (CSES), Ministry of Health Achievement Reports, administrative data, and budget/expenditure reports. The study focuses on 2019 as 2020 data is not likely to fairly represent the efficiency of the public health system due to pandemic-related changes in health care-seeking behavior. In addition, 2019 provides a baseline to enable the future performance evaluations of the impact of the D&D policy change.

This analysis aligns, to the degree possible, with the major social health insurance reimbursement categories at the hospital and health center levels (see Table 1). Due to administrative data limitations, expenditures by facility type are estimated using National Health Accounts data weighted according to the number of each facility type by province [9]. To avoid double counting, maternity services (i.e. delivery and abortion care) are subtracted out from outpatient cases for health centers and inpatient cases for hospitals by province. We convert Khmer Riel (KHR) to United States dollars (US$) using the standard rate 4100 KHR = 1US$.

Technical efficiency can be assessed in terms of the amount or mix of service outputs that can be produced within a given budget [7]. Data Envelopment Analysis (DEA) is a performance benchmarking method in operations management based on a systems view of production according to which resources/inputs are processed into products/outputs. The resulting metric is a measure of technical efficiency relative to the most efficient decision-making units (DMU) or unit of analysis which form the best practice or efficiency frontier. A fundamental assumption behind this method is that if a given producer is capable of producing X units of output with Y inputs, then other producers should also be able to do the same if they were to operate efficiently [36]. DEA is an established performance measurement method and has been used to evaluate the technical efficiency of health systems ([8, 37–42]. The approach can use the radial projection to the efficiency frontier to calculate target outputs levels for each inefficient DMU [43–45].

This study used STATA 17 to calculate all efficiency metrics and associated Simar-Wilson regression modeling [46, 47]. Technical efficiency measures are overly optimistic under standard underlying DEA assumptions [46]. Therefore, we use bootstrapping (2000 times of repeated sampling, with α = .05) to estimate bias-corrected technical efficiency scores. This approach uses Debreu-Farrell output distance functions and enables the calculation of lower and upper bounds [48, 49]. Given the Royal Government of Cambodia’s (RGC) current D&D reform program discussed above, the unit of analysis or DMU is the provincial-municipal administration.

We fit two DEA output-oriented models: (1) hospital services, and (2) health center services. The nonparametric test of returns to scale indicated variable returns to scale (VRS) for the hospital model and constant returns to scale (CRS) for the health center model. The input for each model is the summed total of expended financial resources including staff salaries, pharmaceuticals and consumables, equipment and supplies, other operating costs, social health insurance service payments (from the Health Equity Funds and the National Social Security Fund), and performance-based service delivery grants. Service delivery grants provide public health facilities with additional, flexible budget for delivering quality health services while incentivizing quality improvement [50]. Outputs focus on the major social health insurance payment categories (i.e. outpatient cases, inpatient cases, major and minor surgeries, and maternity care). We limited the input and output factors to the essential components of the service production process to improve the discriminatory power of the analysis given the number of DMUs (*N* = 25) [51]. The hospital model excludes national level hospitals from the municipality of Phnom Penh as they provide specialized services which are not comparable with services provided by CPA1–3 hospitals (see Table 1). Table 2 presents descriptive statistics for all variables used in the DEA models.

**Table 2:** Descriptive statistics for inputs and outputs for the hospital and health center services models

	Sum	Mean	Median	SD	Min	Max
	(1)	(2)	(3)	(4)	(5)	(4)
**Hospital Services Model**						
**Inputs (US$)**						
Staff salaries	46,859,986	1,874,399	1,768,218	852,593	566,232	3,530,746
Pharmaceuticals and consumables	739,853	29,594	12,961	32,150	0	112,171
Equipment and supplies	609,012	24,360	17,381	26,978	0	104,544
Other operating costs	27,081,818	1,083,273	996,914	437,520	521,485	1,953,461
SHI Service Payments	19,349,376	773,975	381,258	1,699,779	21,727	8,823,583
Service Delivery Grants	10,563,269	422,531	368,828	245,809	100,841	954,413
Total Hospital Inputs	105,203,314	4,208,133	3,444,580	2,712,180	1,455,698	14,810,350
**Outputs**						
Outpatient cases	1,762,958	70,518	38,434	73,304	933	319,679
Inpatient cases	577,938	23,118	21,045	13,422	2094	48,930
Major surgeries	51,840	2074	1375	2135	0	8827
Minor surgeries	38,880	1555	1125	1524	0	6597
Maternity services	123,747	4950	4556	2978	309	10,536
**Health Center Services Model**						
**Inputs (US$)**						
Staff salaries	46,919,583	1,876,783	1,673,185	1,218,450	90,173	3,959,417
Pharmaceuticals and consumables	695,758	27,830	13,397	35,701	0	137,128
Equipment and supplies	475,329	19,013	12,780	20,258	0	69,390
Other operating costs	26,363,296	1,054,532	904,118	718,193	98,746	2,594,306
SHI Service Payments	16,701,444	668,058	324,696	1,075,967	3287	5,508,723
Service Delivery Grants	10,873,438	434,938	382,482	333,136	15,257	1,162,660
Total Health Center Inputs	102,028,848	4,081,154	3,519,662	2,774,032	220,243	9,246,370
**Outputs**						
Outpatient cases	9,001,900	360,076	267,371	302,626	19,092	1,058,099
Inpatient cases	57,694	2308	519	3220	0	11,457
Maternity services	140,979	5639	5911	3518	198	12,816

SD = Standard Deviation, US$ = United States dollars, SHI = Social Health Insurance.

The second stage analysis employs truncated regression using the Simar and Wilson approach (weighted by the number of hospitals or health centers for each province-municipality) to assess explanatory factors of the bias-corrected technical efficiency scores [52]. The results for both models were separately regressed on population size, hospital and health center quality scores (and their respective quadratic terms), number of large and small private providers (and the quadratic term for the health center model) as well as hospital and health center non-discretionary resources. The quality score is a facility-level index based on the three healthcare quality dimensions of structure, process, and outcome; scores are collected every 3 months through a national monitoring system [50]. Non-discretionary resources are defined as the summed total of staff salaries, pharmaceuticals and consumables, equipment and supplies, and other operating costs. The health center regression model also included hospital and health center utilization rates. The hospital and health center regression models are shown in eqs. 1 and 2, respectively. Descriptive statistics for explanatory variables used in the models are presented in Table 3.

1 $$ Hospital\_ Technical\_{Efficiency}_i={\mathrm{B}}_0+{\mathrm{B}}_1{\mathrm{Population}}_{\mathrm{i}}+{\mathrm{B}}_2\mathrm{Hospital}\_{\mathrm{quality}}_{\mathrm{i}}++{\mathrm{B}}_3\mathrm{Hospital}\_\mathrm{quality}\_{\mathrm{squared}}_{\mathrm{i}}\ {\mathrm{B}}_4\mathrm{Large}\_\mathrm{private}\_{\mathrm{providers}}_{\mathrm{i}}+{\mathrm{B}}_5\mathrm{Discretionary}\_\mathrm{resources}\ {\left(\mathrm{logged}\right)}_{\mathrm{i}}+{\mathrm{B}}_6\mathrm{Nondiscretionary}\_\mathrm{resources}\ {\left(\mathrm{logged}\right)}_{\mathrm{i}}+{\upvarepsilon}_{\mathrm{i}} $$

2 $$ Health\_ Center\_ Technical\_{Efficiency}_i={\mathrm{B}}_0+{\mathrm{B}}_1{\mathrm{Population}}_{\mathrm{i}}+{\mathrm{B}}_2\mathrm{Hospital}\_{\mathrm{utilization}}_{\mathrm{i}}+{\mathrm{B}}_3\mathrm{Health}\_\mathrm{center}\_{\mathrm{utilization}}_{\mathrm{i}}\ {\mathrm{B}}_4\mathrm{Health}\_\mathrm{center}\_{\mathrm{quality}}_{\mathrm{i}}+{\mathrm{B}}_5\mathrm{Health}\_\mathrm{center}\_\mathrm{quality}\_{\mathrm{squared}}_{\mathrm{i}}+{\mathrm{B}}_6\mathrm{Small}\_\mathrm{private}\_{\mathrm{providers}}_{\mathrm{i}}+{\mathrm{B}}_7\mathrm{Small}\_\mathrm{private}\_\mathrm{providers}\_{\mathrm{squared}}_{\mathrm{i}}+{\mathrm{B}}_8\mathrm{Discretionary}\_\mathrm{resources}\ {\left(\mathrm{logged}\right)}_{\mathrm{i}}+{\mathrm{B}}_9\mathrm{Nondiscretionary}\_\mathrm{resources}{\left(\mathrm{logged}\right)}_{\mathrm{i}}+{\upvarepsilon}_{\mathrm{i}} $$

**Table 3:** Descriptive statistics of explanatory variables used in the second stage analysis (2019 data)

	Sum (1)	Mean (2)	Median (3)	SD (4)	Min (5)	Max (6)
Population (both models)	16,341,870	653,675	634,448	477,239	42,516	1,861,611
**Hospital Services Model**						
Hospital quality scores (mean, weighted)	–	65.8	67.1	12.6	40.6	85.0
Large private health providers	841	34	11	86	1	439
Discretionary resources (US$)	29,912,645	854,374	620,033	849,327	120,042	4,420,271
Nondiscretionary resources (US$)	75,290,669	3,011,627	2,764,244	1,220,138	1,173,043	5,374,128
**Health Center Services Model**						
Hospital utilization rate (per 1000)	–	60	57	2.6	1	11.3
Health center utilization rate (per 1000)	**–**	553	518	208	141	880
Health center quality scores (mean)	–	69.1	70.3	10.9	44.7	92.0
Small private health providers	13,734	549	482	499	47	2272
Discretionary resources (US$)	27,574,882	1,103,995	835,136	1,120,748	18,544	5,891,205
Nondiscretionary resources (US$)	74,453,966	2978,159	2,596,936	1,906,678	201,699	6,025,001

Note: SD = Standard Deviation

The exploratory analysis tested other variables that could potentially explain technical efficiency including population density, proportion of urban/rural population, poverty density, mean days of productively loss due to illness or injury, distance from the capital city Phnom Penh, education completion rate, literacy rate, and the proportion of women citing distance as a barrier to accessing healthcare. Quadratic and logarithmic transformations for each variable were also tested; these variables (along with hospital and health center utilization rates in the hospital model) were excluded from the final regression models as they did not add explanatory power nor improve the fit as evaluated using the log ratio test [53]. To test for sensitivity to outliers, the final models were run with and without outliers. All units of analysis were retained as this did not change the direction nor significance level of the results.

Finally, we use RStudio 4.1.0 (R [54]) to estimate the unit cost for each of the major insurance reimbursement categories by using the DEA Aumann-Shapley applied cost allocation approach. The Aumann–Shapley prices associated with a given output vector are estimated by weighting the sum of gradients of the linear facets of the estimated cost function along a radial contraction path of the observed output vector. The weights are proportional to the length of the projected line segments [55].

Calculations are proportionally weighted and restricted (+/− 50%) from updated Deutsche Gesellschaft für Internationale Zusammenarbeit (GIZ) Social Health Protection Programme costing data that was compiled using a standard step-down micro-costing methodology for public health centers and referral hospitals CPA1–3 [13, 31]. Hence, we use the GIZ weights as a reference, but allow for deviations of the relative GIZ price 50% up and down. We estimate a VRS (hospitals) and CRS (health centers) cost functions C(.) with restrictions on the dual weight. Equation 3 expresses the assumption of the ratio of the dual weights for *k* outputs.

3 $$ 0.5\ast \frac{GI{Z}_j}{GI{Z}_1}\le \frac{v_j}{v_1}\le 1.5\ast \frac{GI{Z}_j}{GI{Z}_1}\  for\ j=2,\dots, k $$

Using the estimation technology, we calculate the fixed costs C (0). The efficient variable costs *C*^∗^(*y*) = *C*(*y*) − *C*(0) can now be allocated using Aumann-Shapley (A-S) prices. The theoretical literature has shown this method (and A-S prices) possesses a number of desirable properties, and it has essentially been the unanimous recommendation of economists for decades when sharing the costs of joint production [56]. The A-S price of product j is the average marginal cost as shown in eq. 4.

4 $$ {p}_j={\int}_0^1{\delta}_jC(ty) dt\simeq \frac{1}{S}\sum \limits_{s=1}^SM{C}_j\left(\frac{s}{S}y\right) $$

Here S is the number of steps we use in the approximation and $M{C}_j\left(\frac{s}{S}y\right)$ is the marginal costs of product j at the point $\frac{s}{S}y$. Using these prices, we can allocate a large share of the cost of a DMU. What is left is the fixed costs and the possible inefficiency (see eq. 5).

5 $$ RemainingCosts= ActualCosts-\sum \limits_{j=1}^{k\ }{p}_j{y}_j $$

We allocate the non-allocated, remaining costs proportional to the allocated cost shares. Hereby the final costs assigned to product j is calculated following eq. 6.

6 $$ {p}_j{y}_j+ Remaining\ Costs\frac{p_j{y}_j}{\sum \limits_{j=1}^{k\ }{p}_j{y}_j} $$

As hospital data was aggregated by province-municipality, hospital level (i.e. CPA1–3) specific rates are estimated using weights calculated by averaging the social health insurance payment rates and costing data described above. We consider this to be a rational approach as there is evidence that total and unit costs of different hospital levels are not significantly different [31].

Finally, we use service-specific facility level utilization rates, output production targets calculated from the bias-corrected technical efficiency scores, and unit costs calculated from the applied cost allocation to estimate the potential additional social health insurance beneficiaries that could be enrolled and served by the public health system with existing supply-side financing (i.e. exempting user fee reimbursement payments) if the system were operating efficiently. We account for new beneficiary enrolment in 2020 under the On-demand ID Poor system noted above, and assume a five (5) percent service utilization increase for both current and the potential newly enrolled beneficiaries.

## Results

**Fig. 1:**
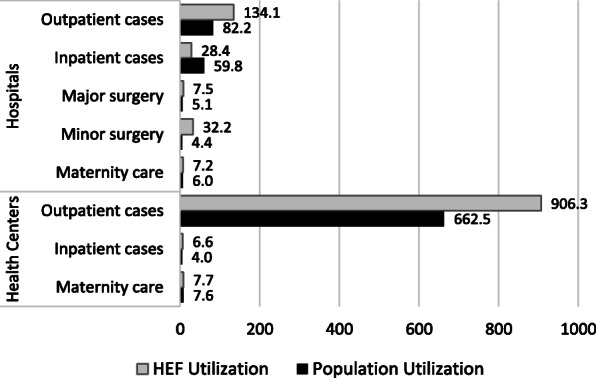
Comparison of hospital and health center utilization rates per 1000 among Health Equity Fund (HEF) beneficiaries and the general population (2019 data)

Figure 1 compares hospital and health center service utilization for each output-payment category. With the exception of hospital inpatient cases, HEF beneficiaries generally used public health services at a higher rate compared to the rest of the population (i.e. non-inclusive of HEF beneficiaries) for each major service category. However, inpatient cases at the health center level among HEF beneficiaries are 1.63 times higher (6.6/4.0) compared to the rest of the population. Outpatient cases are 1.63 and 1.37 times higher compared to the rest of the population, at hospitals and health centers, respectively. In addition, minor surgeries at hospitals are 7.3 times higher than for non-HEF beneficiaries.

**Fig. 2:**
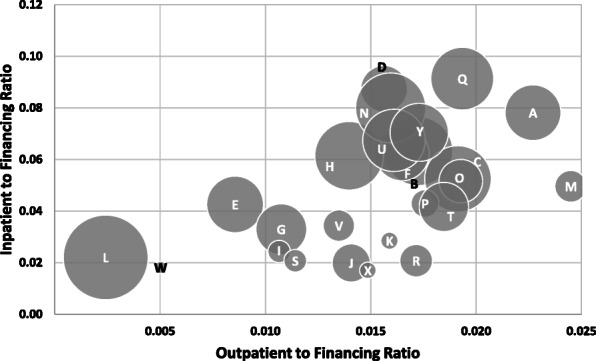
Scatterplot of inpatient days and outpatient services to total financing ratios by province-municipality, circle size weighted by population size (2019 data)

Figure 2 shows the total hospital and health center inpatient and outpatient to financing ratios for each province-municipality. Lower ratio values indicate lower inpatient and outpatient outputs relative to the inputs. The variation among provinces highlights the different output levels given their respective inputs. Provinces L and W are outliers in both their relative output to financing ratios and their population sizes. However, provinces X and K are similar in size to W and have notably higher outpatient to financing ratios. Smaller population provinces tend to have lower inpatient to financing ratios, but are comparable to other provinces in relation to outpatient to financing ratios.

Table 4 summarizes the technical efficiency (TE) results. The mean population weighted efficiency scores are 1.12 (hospitals) and 1.41 (health centers); the mean bias-corrected, population weighted scores are 1.34 (hospitals) and 1.73 (health centers). This suggests that, overall, if the public health system were fully efficient, hospital service output could increase by 34%, and health center service output could increase by 73% with current financing. Kep was found to be an outlier with three times the ratio of bias squared to variance for its radial measure less than 1. Thus, bias-corrected scores were not calculable for this province. A scatterplot of hospital and health center TE scores by province-municipality is presented in appendix 1.

**Table 4:** Hospital and health center services output-oriented Debreu-Farrell efficiency scores with 95% confidence limits for 25 provincial-municipal administrations (2019 data)

		Hospital Services Model				Health Center Services Model			
DMU code	Province-Municipality	TE	Bias-corrected TE	TE Lower Limit	TE Upper Limit	TE	Bias-corrected TE	TE Lower Limit	TE Upper Limit
(1)	(2)	(3)	(4)	(5)	(6)	(7)	(8)	(9)	(10)
A	Banteay Meanchey	1.28	1.52	1.36	2.00	1	1.08	1.00	1.29
B	Battambang	1.01	1.19	1.05	1.65	1.15	1.20	1.16	1.27
C	Kampong Cham	1	1.36	1.10	2.43	1.57	1.63	1.58	1.78
D	Kampong Chnang	1	1.63	1.42	5.19	1.01	1.05	1.01	1.18
E	Kampong Speu	1.20	1.33	1.24	1.51	1.18	1.21	1.18	1.32
F	Kampong Thom	1.05	1.16	1.09	1.43	1.09	1.13	1.09	1.22
G	Kampot	1	1.14	1.04	1.31	1.34	1.38	1.34	1.50
H	Kandal	1	1.14	1.05	1.37	1.23	1.29	1.24	1.46
I	Koh Kong	1.84	2.14	1.95	2.96	2.06	2.14	2.08	2.26
J	Kratie	1.27	1.38	1.30	1.58	1.45	1.50	1.45	1.68
K	Mondulkiri	1.69	2.10	1.81	3.44	1.04	1.12	1.05	2.12
L	Phnom Penh	1.31	1.44	1.32	1.81	2.90	3.01	2.93	3.24
M	Preah Vihear	1	1.35	1.11	2.15	1	1.22	1.01	4.15
N	Prey Veng	1.09	1.21	1.12	1.39	1.06	1.12	1.07	1.30
O	Pursat	1.09	1.19	1.11	1.41	1	1.06	1.01	1.13
P	Rattanakiri	1.01	1.19	1.06	1.94	1.38	1.48	1.40	2.53
Q	Siem Reap	1	1.44	1.19	2.77	1.18	1.24	1.19	1.42
R	Sihanoukville	1	1.24	1.08	2.00	1.11	1.14	1.12	1.24
S	Stung Treng	2.41	2.65	2.47	3.32	1.29	1.34	1.29	1.45
T	Svay Rieng	1	1.11	1.05	1.26	1.30	1.36	1.31	1.47
U	Takeo	1	1.37	1.15	2.41	1.43	1.49	1.44	1.65
V	Oddor Meanchey	1	1.26	1.07	2.66	1.66	1.73	1.68	1.87
W	Kep	1	.	.	.	1.64	1.71	1.64	1.99
X	Pailin	1	1.68	1.72	7.99	1.06	1.09	1.06	1.18
Y	Tbaung Khmoum	1.27	1.44	1.29	1.80	1.07	1.11	1.07	1.23
	**Min**	1	1.11	1.03	1.26	1	1.05	1	1.13
	**Max**	2.41	2.65	2.47	7.99	2.90	3.01	2.92	4.15
	**Mean**	1.18	1.45	1.30	2.41	1.32	1.39	1.34	1.72
	Weighted Mean*	1.12	1.34	1.19	1.36	1.41	1.73	1.66	2.13
	**Median**	1.01	1.35	1.14	1.97	1.18	1.24	1.19	1.46

Notes: DMU = Decision Making Unit, TE = Technical Efficiency, *weighted by population,

The correlation coefficient of the bias-corrected hospital and health center scores is 0.16. This suggests a positive, but minimal relationship between hospital and health center efficiency.

Table 5 presents results from the Simar Wilson regressions of the bias-corrected, Shephard distance technical efficiency scores. Population size has a positive, highly statistically significant effect on hospital efficiency, but not health center efficiency. Both the hospital and health center models reveal health service quality, private providers, and non-discretionary health resources/financing to be statistically significant factors affecting technical efficiency.

**Table 5:** Explanatory factors for hospital and health center technical efficiency (2019 data)

VARIABLES	Hospital Services Model (1)	Health Center Services Model (2)
Population (per 100,000)	0.033*	−0.003
	(0.015)	(0.013)
Hospital quality scores	−0.050*	
	(0.020)	
Hospital quality scores (squared)	0.000*	
	(0.000)	
Large-scale private healthcare providers	−0.001**	
	(0.000)	
Hospital discretionary resources (logged)	0.055	
	(0.071)	
Hospital non-discretionary resources (logged)	−0.241	
	(0.139)	
Hospital utilization rate (per 1000)		−0.006***
		(1.421)
Health Center utilization rate (per 1000)		0.000***
		(0.123)
Health center quality scores		−0.058*
		(0.029)
Health center quality scores (squared)		0.000
		(0.000)
Small-scale private healthcare providers		0.001*
		(0.000)
Small-scale private healthcare providers (squared)		−0.000***
		(0.000)
Health center discretionary resources (logged)		0.074
		(0.064)
Health center non-discretionary resources (logged)		−0.241*
		(0.096)
sigma	0.078***	0.064***
	(0.013)	(0.010)
Constant	5.048*	5.501***
	(2.235)	(1.045)
Observations	116	1222
Wald Chi2	18.85	81.56

Standard errors in parentheses.

*** p < 0.001, ** p < 0.01, * p < 0.05

**Fig. 3:**
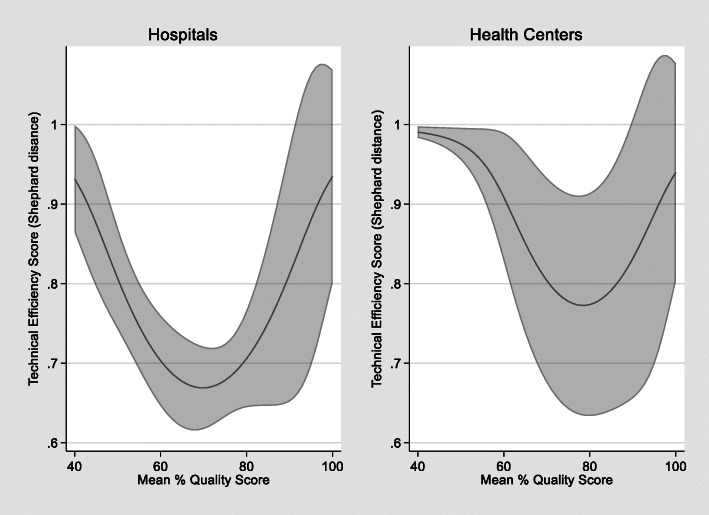
Predicted marginal effects of provincial-municipal level mean quality scores on hospital and health center technical efficiency with 95% confidence intervals (2019 data)

In relation to health facility quality scores, there are statistically significant, non-linear effects on technical efficiency both at the hospital and health center levels. Figure 3 shows the predicted marginal effects of healthcare quality scores on technical efficiency after controlling for population size and other model covariates. The result shows that provinces with the lowest and highest quality scores have higher technical efficiency. However, technical efficiency increases with quality scores once the score reaches a critical threshold of about 70% for hospitals and 80% for health centers.

**Fig. 4:**
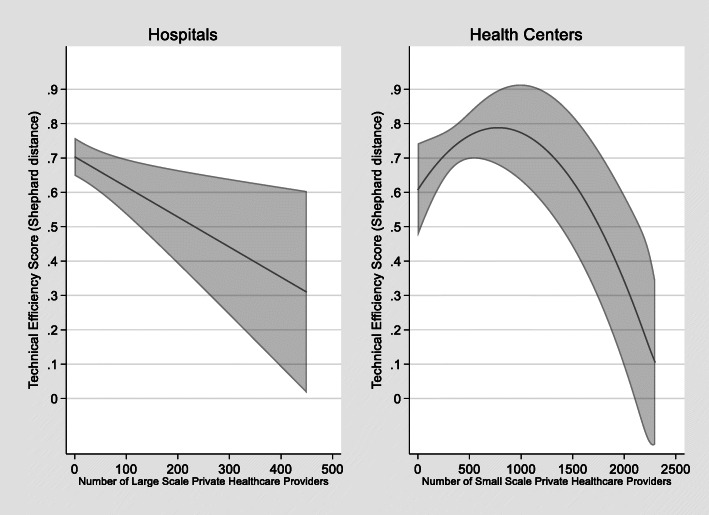
Predicted marginal effects of provincial-municipal level private providers on public hospital and health center technical efficiency with 95% confidence intervals (2019 data)

In addition, the number of private sector providers has a statistically significant effect on public health facility technical efficiency, both at the hospital and health center levels. Figure 4 shows the predicted marginal effects of private healthcare providers on public hospital and health center technical efficiency after controlling for population size and other covariates. For hospitals, there is a small (− 0.001), but statistically significant (*p* < 0.01) negative effect on technical efficiency as the number of large-scale private healthcare increases. For health centers, there is a non-linear effect with small (0.001), but statistically significant (*p* < 0.05) positive effect on technical efficiency when the number of small-scale private providers is limited – up to about 750. However, beyond this threshold additional private providers have a small, but highly statistically significant (*p* < .001) negative effect on health center technical efficiency.

In addition, the analysis shows that a 1% increase in hospital non-discretionary resources decreases hospital technical efficiency by 0.241 points (*p*-value< 0.08). The same effect is observed for health centers: a 1% increase in non-discretionary resources decreases technical efficiency by 0.241 points (p-value< 0.05). Finally, the hospital utilization rate has a highly statistically significant (*p* < 0.001) negative effect on health center technical efficiency.

Table 6 compares the social health insurance public health facility payment rates (columns 1–2), Jacobs et al. costing study results and results from the continued data collection from the GIZ study (columns 3–4). Column 5 shows the adjusted GIZ costing data; the adjustment was done to account for the difference between the total cost in the study data (i.e. US$ 105,203,311 for hospitals and US$102,028,845 for health centers) and the estimated cost assuming the GIZ service specific unit costs multiplied by total service outputs.^1^ For the hospital model the total cost in the data only accounted for 67.2% of the projected (GIZ) costs (i.e. US$156,608,043). For the health center model the total cost in the data was 209% of the projected (GIZ) costs (i.e. US$48,828,997) indicating that the GIZ costs can only explain about half of the actual costs. The Aumann-Shapley estimates from this study (column 6) are presented for each facility level and major service category. Column 7 shows the relative difference between the Aumann-Shapley estimates and the adjusted GIZ costing data estimates, i.e. the difference expressed as a ratio of the GIZ costing data estimates. Service costs at CPA1 hospitals are higher compared to CPA2 hospitals and to a lesser degree CPA3 hospitals. This is attributed to low service volume [13].

**Table 6:** Social health insurance public health facility payment rates and costing study results by facility level and major service category in US$

	HEF Payments	NSSF Payments	[13]	GIZ costing data, 2019	GIZ costing data, 2019 adjusted	Current study	Relative difference
**CPA3 Hospital**	(1)	(2)	(3)	(4)	(5)	(6)	(7)
Outpatient cases	7.80	7.80	41.53	19.51	13.11	20.15	0.54
Inpatient cases	29.27	40.73	158.21	169.48	113.85	157.32	0.38
Maternity care	19.51	37.80	46.57	39.18	26.32	64.31	1.44
Major Surgery	243.90	243.90	29.79	43.88	29.48	149.90	4.09
Minor Surgery	97.56	48.78	34.04	38.78	26.05	38.26	0.47
**CPA2 Hospital**							
Outpatient cases	3.90	3.90	5.87	7.76	5.21	5.63	0.08
Inpatient cases	24.39	28.78	86.53	100.99	67.84	95.21	0.40
Maternity care	19.51	29.27	27.75	44.93	30.18	54.60	0.81
Major Surgery	78.05	97.56	24.87	87.80	58.98	76.96	0.30
Minor Surgery	48.78	48.78	25.87	31.57	21.21	27.06	0.28
**CPA1 Hospital**							
Outpatient cases	2.44	2.93	9.65	16.17	10.86	8.20	−0.25
Inpatient cases	19.51	31.71	291.45	129.74	87.15	186.88	1.14
Maternity care	19.51	24.39	66.72	51.98	34.92	73.09	1.09
Major Surgery	n. a.	n. a.	n. a.	n. a.	n. a.	n. a.	n. a
Minor Surgery	39.02	24.39	40.39	39.24	26.36	37.45	0.42
**Health Center**							
Outpatient cases	0.98	1.46	3.88	3.74	7.81	6.51	−0.17
Inpatient cases	19.51	19.51	12.46	15.78	32.97	32.98	−0.05
Maternity care	19.51	19.51	107.29	101.09	211.23	344.00	0.63

HEF = Health Equity Funds; NSSF = National Social Security Funds

Aumann-Shapley applied cost allocation estimates for each major social health insurance payment category are comparable to the updated GIZ costing data with a few notable exceptions. GIZ data shows major surgery at CPA3 hospitals to be about half the cost of major surgery at CPA2 hospitals, $43.88 and $87.80 respectively, (unadjusted) and $29.48 and $58.98 (adjusted) respectively. Aumann-Shapley estimates show major surgery at CPA3 hospitals to be about double the cost of major surgery at CPA2 hospitals, $149.90 and $76.96 respectively. Likewise, GIZ data shows health center maternity service cost to be higher ($101.09) than CPA1–3 hospital maternity costs, $51.98, $44.93, and $39.18 respectively. The corresponding adjusted rates are $211.23 for maternity care at health centers and $34.92, $30.18, and $26.32 at hospitals CPA1–3 respectively.

Aumann-Shapley health center maternity service costs are substantially higher ($344.00) compared to GIZ costing data. This can be attributed to the fact that there are additional service categories such as chronic patient care which were excluded from the DEA model as utilization and case count data were not available. Therefore, costs for these unaccounted-for services are redistributed to other service categories which increases the health center cost estimates for maternity services and makes them higher compared to the GIZ costing estimates.

Health Equity Fund payments cover between 6% and 255% of the estimated unit costs, corresponding to health center maternity care and CPA3 hospital minor surgery respectively. Most notably, minor and major surgery payments exceed the estimated unit costs for these services. HEF payments as a proportion of the Aumann-Shapley costs by facility level and service category are presented in appendix 2.

**Fig. 5:**
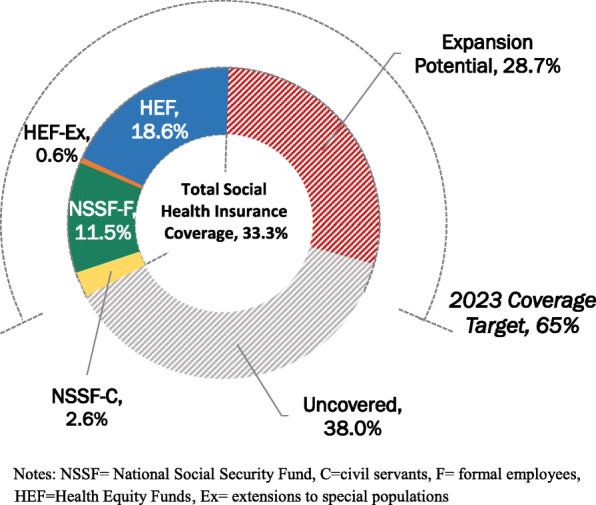
Effective social health insurance coverage, expansion potential (2021 estimates), and target coverage

Figure 5 shows the current coverage of the four social health insurance schemes in relation to the expansion potential and national target. Using facility-level efficiency scores, unit costs, and service utilization data, we estimate the potential supply-side ‘service space’ to expand population coverage with existing supply-side financing if the public health system were fully efficient. This equates to an additional 4.69 million new social health insurance beneficiaries or about 28.7% of the population. The potential expansion would make a substantial contribution towards reducing the population coverage gap towards Cambodia’s National Strategic Development Plan coverage target of 65% of the population by 2023.

## Discussion

There are several limitations to this study. First, the potential population coverage expansion estimates and associated increased service utilization related to increased technical efficiency assume that public facility user-fees will be paid under a social health insurance scheme. The projected cost of additional user-fee payments to expand the Health Equity Fund to the uncovered 1st–3rd wealth quintile people (approximately 3.5 million people) are modeled to range from US$ 23.0 (using current HEF payment rates) to 36.5 million (using NSSF payment rates), assuming the adoption of several complimentary policy options [17].

Second, the study does not assess performance relating to high-level health outcomes such as morbidity, mortality or life-expectancy. However, given the overall low utilization of public health services and the plethora of factors which impact on such outcomes, the public health system’s contribution is likely limited and very difficult to measure. In addition, there is very limited data available that measure such outcomes at the provincial-municipal level.

Third, this study does not evaluate the efficiency of private sector services which are the predominant provider as comparable data is not available. However, the study does assess the impact of private providers on public health service efficiency by including several related variables in the second stage analysis. In addition, this study does not assess technical efficiency among individual public health facilities. Although this approach is possible if the data is available our focus is on the provincial-municipal level where management responsibility has recently been delegated. The study estimates grouped hospital and grouped health center technical efficiency at the provincial-municipal level. With the exception of specialized national hospitals, this approach captures the full range of public health services and aims to provide a better understanding of where to focus efficiency improvement efforts within each province.

Fourth, we find that for public health facilities to be fully efficient service output would need to increase by 34% and 73% for hospitals and health centers, respectively. It is important to note that DEA is only appropriate to compare like units (i.e. aggregated hospital services are compared with other aggregated hospital outputs and aggregated health center services with other aggregated health center services). Therefore, the result cannot be interpreted to mean that the primary care services provided by Cambodian public health centers are less cost-effective compared to Cambodian public hospitals. Rather, the results of this study show that in Cambodia there is more variation in technical efficiency among health center service output grouped at the province-municipality level (compared to hospitals) and therefore more opportunity to increase outputs and service space for the services they provide.

Fifth, comparative service-type unit costs do not exactly align with the social health insurance payment categories. We address this issue by generalizing unit costs which include the specific reimbursement category. In addition, published unit cost data is limited to health facilities in three provinces. To increase the reliability of the estimates we also compare unit cost estimates with updated data from ongoing data collection of a high-quality costing study. In addition, the model outputs are limited to the major service categories. Two additional service payment categories (i.e. emergency services and long-acting family planning methods) were initially included in the models as these services have different payment rates (with utilization/claims data recorded by the social health insurance mechanisms). However, the Ministry of Health does not separate these services in the provincial-municipal aggregated data and the assumptions required to model utilization for these services among the general population yielded inconsistent results. In addition, the discriminatory power of DEA is constrained when there is a large number of inputs and outputs and a small number of decision-making units. Limiting the model to the essential components of the service production process is considered a best practice [51].

Finally, this study does not assess system-wide reforms that could further improve cost efficiency or financial savings such as pooling health insurance funds and merging schemes, improving procurement to lower the purchase cost of pharmaceuticals, consumables, equipment and supplies, and reducing overhead [6, 57]. Such measures could increase budgetary space for health providing that they are well-defined and public financial management systems enable such gains to be repurposed toward prioritized health needs [58]. This topic is further discussed below.

This study assesses public health service technical efficiency at the provincial-municipal level. The results reconfirm under-utilization of public health services and quantify the potential to improve efficiency by expanding social health insurance population coverage with current supply-side financing. These findings are consistent with other empirical studies. Ensor et al. found HEF to be associated with higher public health facility efficiency [11]. A recent costing study found that most health facilities make a minor surplus suggesting that they could increase the number of patients without running a loss [31]. Jacobs et al. note that service volume along with contextual factors such as poverty incidence, population density and accessibility affect unit costs [13].

There is mixed evidence as to if the HEF increases public health service utilization due to issues with gaps in financial risk protection, general low utilization of public providers, and deficient eligibility targeting [10]. However, a direct comparison of utilization rates by service level and type demonstrates that public health service utilization among HEF beneficiaries is generally higher compared to the rest of the population. This provides evidence that Cambodia’s largest social health protection scheme improves access. Notwithstanding, it is important to note that utilization data does not capture effectiveness or quality of the service provided [59].

There is some evidence that fee-for-service reimbursements, the system used by Cambodia’s social health protection schemes, may contribute to oversupply as it incentivizes service provision [60–63]. However, provider remuneration is complex and there is also evidence that the risk of overprovision is contextual ([64, 65]. Moreover, health service utilization rates in Cambodia are considered low compared with other Asian countries [66].

This study estimates the potential supply-side ‘service space’ for 4.69 million additional social health protection beneficiaries in a fully efficient public health system. This could raise total population coverage of social health insurance to 60% while leveraging the unutilized service capacity of the public health system. However, this still leaves a population coverage gap of 40%. The gap is worrisome given the expected decline in out-of-pocket spending on healthcare due to pandemic-related economic hardship which will need to be offset with public financing [67]. Additional investments in the health system can ensure access to needed health services, particularly among the financially vulnerable [17]. For Cambodia this would imply an increase in government health expenditure of 0.6% of GDP [67]. Although policymakers may raise concerns about adding budget to an inefficient system, there are several smart investments to promote continuous health system efficiency improvement. These include the prioritization of primary health care, strategic purchasing, alignment of financing and delivery, better accountability through results-based outcome and output contracts and related provider incentives, decentralization, moving care out of hospitals, and independent regulatory agencies [57, 58, 68–70]. For example, one simple measure would be to link all social health insurance provider payments to both service provision and health facility quality scores [50]. Evidence shows that government health expenditure as a percentage of total health expenditure (i.e. inclusive of out-of-pocket expenditure) is positively associated with efficiency [71]. The expansion of social health protection, particularly to the financially vulnerable, can support economic recovery by enabling households to maintain productivity, thereby stabilizing household income and expenditure.

Finally, efficiency gains need to be reinvested to provide an incentive for continuous health system performance improvement [58]. To effectively address public health service inefficiency, provincial-municipal administrations need to be given adequate flexibility to reallocate resources to increase the volume or quality of the most efficiently delivered services [58]. In addition, predictable financing to sub-national governments is imperative to improve health service performance (Gertler, Giovagnoli and Martinez 2014).

The second stage analysis identifies several factors which explain the variation in technical efficiency among the provinces. High service volume hospitals are generally considered to be associated with better outcomes and economies of scale [72]. However, this study did not find utilization rates to be a significant explanatory factor of hospital efficiency. This may be attributable to negative spillover effects whereas increased volume in one service area may be associated with increased cost in another area [73]. By contrast, health centers offer a much more limited range of services, and therefore less potential for negative spillovers. This study did find a small, but highly significant positive relationship between health center utilization and technical efficiency.

The finding that provinces with the lowest and highest quality scores have higher technical efficiency suggests that among provinces with lower health facility quality scores, some improvements in quality may decrease technical efficiency, potentially indicating that the initial investments in quality such as training and facility upgrades increase costs and/or decrease service output. Similarly, it could also indicate that facilities with the lowest quality scores are underfinanced and therefore do not invest in quality improvement measures but have high patient volume which yields higher technical efficiency scores. This suggests that provincial-municipal level public health facilities need to attain a quality score critical threshold of about 70%-80% before quality improvement can contribute to technical efficiency. A study of Portuguese public hospitals found that good clinical safety practices tend to be associated with low technical efficiency, concluding that there are trade-offs between efficiency and quality [74].

The nonlinear relationship between small-scale private providers and public health centers suggests that there may be service complementarity between the sectors when the number of private providers is limited. However, the overall marginal negative effect of private sector providers on public health service technical efficiency is likely due to competition which reduces the number of patients seeking public sector care. Moreover, the dominance of the largely unregulated, pro-rich private sector accounts for a significant proportion (57.5%) of out-of-pocket spending [75, 76]. As private health services and health insurers can exacerbate health inequity, it is essential for countries to determine the appropriate level of privatization in their systems which necessitates transparent and responsible regulation alongside efforts to improve public system efficiency [77].

The marginal negative effect of supply-side resources/financing on technical efficiency suggests that increased financial autonomy and demand-side financing may yield better value for money. Health financing should focus on smart investments discussed above such as increasing social health insurance payments.

The finding that the hospital utilization has a large, statistically significant negative effect on health center technical efficiency suggests that patients bypassing health centers and going directly to hospitals is an issue. This is consistent with other research in Cambodia which found that primary care facilities are regularly bypassed due to a lack of key personnel, stock-outs of essential drugs and substandard quality of care [31]. This may be redressed by correcting the underlying causes and incentivizing health center referrals such as prioritizing service provision at hospitals for patients with a formal health center referral.

There are likely additional or secondary factors which contribute to public health system underutilization. For example, systematic factors can lead to patient avoidance of public facilities due to quality perceptions including competency and attitude of providers [78]. Another factor is limited service availability, particularly for non-communicable diseases [10]. Moreover, it is also possible that patients may avoid public care-seeking due to unofficial fees or face substantial indirect financial shocks relating to needed medical care and/or lost productivity [20].

We calculate unit costs using the DEA Aumann-Shapley applied cost allocation approach. To the best of our knowledge, this study is the first time this method has been used for health services costing. The results are comparable with recent, high-quality public health facility costing data, and we believe this approach to be a good alternative to traditional costing studies which can be labor intensive, time consuming, and expensive.

The approach is not without limitations however. First, the number of costing categories is limited as a function of the DEA model. This issue could be mitigated to a degree by increasing the number of decision-making units. For example, if data is available, more robust results would be expected by using hospitals and health centers as the primary unit of analysis (as opposed to grouping them by province-municipality). Second, this Aumann-Shapley analysis used previous costing study results for weighting. Although it is common to rely on existing data to parameterize cost models, it requires that such data exist.

Health provider payments can incentivize or de-incentivize particular services. The Health Equity Fund payment rates are inconsistent with the estimated cost of service provision across the major payment categories. The wide variability (6% - 255%) of payments as a proportion of the estimated unit costs suggests that payment rates should be realigned. In particular, the higher than cost reimbursements for hospital surgeries is notable. Given minor surgeries at the hospital level are 7.3 times higher among HEF beneficiaries compared with the rest of the population, the overpayment may be creating a perverse incentive and service overutilization. In fact, there is evidence of both public and private healthcare facilities providing surgeries for commercial interest [79].

## Conclusions

Many countries have committed to achieving universal health coverage, however pandemic-related decreases in revenue constrain increases to domestic health financing. To advance UHC progress, countries should also focus on efficiency improvements. In Cambodia, public health service efficiency can be improved by increasing utilization of cost-effective services. This can be achieved by enrolling more beneficiaries into the social health insurance schemes with current supply-side financing levels. Other factors that can lead to increased efficiency are improving health service quality, regulating private sector providers, focusing on discretionary financing, and incentivizing a referral system to improve gatekeeping. In addition, the current social health insurance payment rates are not well aligned with the service unit costs. Moreover, shifting financial resources to smart investments, especially demand-side financing including increasing the payment/reimbursement rates would likely further incentivize increased service provision and improve technical efficiency.

## Data Availability

The datasets during and/or analysed during the current study available from the corresponding author on reasonable request.

## Appendix 1

### Scatterplot of hospital and health center technical efficiency by province-municipality, circle size weighted to population size

Shephard distance efficiency scores are the reciprocal of Debreu-Farrell; Shepard distance operates on a scale of 0–1 with one representing (relative) optimal efficiency [36, 79]. Figure 3 shows the hospital and health center Shephard efficiency scores. Provinces L, I and S are outliers in terms of population size and technical efficiency. Notably, there are provinces with population sizes similar to I and S that are more efficient such as K and P.

## Appendix 3

### Health services reimbursement rate and cost by comparison by facility level and service type in Khmer Riel

**Table 1:** Health Center-level

	Health care service type description / reimbursement categories			Payment rates		Estimated actual costs
**No.**	**HEF(**a**)**	**NSSF(**b**)**	**GIZ costing (**c**)**	**HEF**	**NSSF**	**GIZ costing**
	**General Outpatient**					
**1**	**General consultation** on diseases or health issues of citizens of all ages related to reproductive health, infectious diseases, non-communicable diseases and other public health issues, as defined in the maximum package of activities for health centers.	MPA outpatient consultations: New and follow-up outpatient consultations at a health center. The consultation services include: interrogation, physical exam, medical education, counseling, consultation booklet, para-clinic services (malaria rapid test and TB smear), treatment and prescribed medicines, follow-up treatment of TB, DOTS, or leprosy.	Prevention contact	4000	6000	27,000
			OPD services patient contact			14,000
			Per inpatient day			14,500
			Chronic patients contact			120,000
		MPA short-term birth control service			10,000	
	Long-term contraceptive methods using IUD or Implant (IUD / Implant)	MPA long-term birth control		20,000	30,000	
	Screening for cervical cancer			20,000		
	**Emergency (whether to refer or not)**					
**2**	First Aid interventions for patients or victims who are at risk of life threatening with vital/danger signs; emergency acts include: Check, monitor and record regular life signs and treatment according to medical conditions as well as arrangements to refer to the referral hospital as necessary.	MPA emergency and referral or non-referral	OPD services patient contact	20,000	20,000	14,000
			Per inpatient day			14,500
		MPA minor surgical activities			12,000	
**3**	**Delivery and abortion/miscarriage**	MPA delivery	Maternity	80,000	80,000	934,000
**4**	**Inpatient department (IPD)**			**80,000***		

*For health centers with inpatient beds (former district hospitals)

**Table 2:** Hospital-level

No.	Health care service type description / reimbursement categories			Referral hospital, level 1			Referral hospital, level 2			Referral hospital, level 3			National hospital/ national center	
	HEF (a)	NSSF (b)	GIZ costing (c)	HEF	NSSF	GIZ costing	HEF	NSSF	GIZ costing	HEF	NSSF	GIZ costing	HEF	NSSF
	***Outpatient Services***													
**1**	Outpatient checkup and consultation (including small surgical cases that refer to suturing, wound dressing, excision....)	Outpatient consultations	Outpatient / patient	10,000	12,000	33,500	16,000	16,000*	31,000	32,000	24,000	100,000	40,000	60,000
		Non-hospitalized minor surgical procedures			20,000			40,000			40,000			100,000
		Short-acting birth control			10,000									
**2**	Contraceptive method using IUD or Implant	Non-hospitalized minor surgical activities		20,000	20,000		20,000	40,000		20,000	40,000		20,000	100,000
**3**	Permanent methods (Vasectomy and tubal ligation)		Surgery per inpatient day			128,000	100,000		123,000	100,000	400,000	163,000	100,000	600,000
	***Inpatient care***													
**4**	Inpatient treatments	Adult general medicine (hospitalization)	General medicine per inpatient day	80,000	100,000	130,000	100,000	120,000	118,000	120,000	160,000	167,000	140,000	400,000
		Hospitalization for gynecology	Per inpatient day		100,000	157,000		150,000	121,000		200,000	172,000		400,000
		Hospitalization for general child and pediatrics	Paediatrics per inpatient day		92,000	122,000		108,000	123,000		128,000	150,000		350,000
		TB	TB inpatient day		160,000	221,000		180,000	322,000		200,000	182,000		300,000
	***Emergency, surgery, and delivery related services***													
**5**	Emergency services	Emergency		250,000	120,000		250,000	240,000		300,000	320,000		320,000	800,000
**6**	Small surgeries	Moderate surgical activity	Surgery per inpatient day	160,000		128,000	200,000	200,000	123,000	400,000	200,000	163,000	400,000	600,000
**7**	Major surgeries	Major surgical interventions					320,000	400,000		1000,000	1000,000		1,200,000	1,500,000
**8**	Birth delivery, abortion / miscarriage / post-abortion / miscarriage care	Delivery	Maternity per inpatient day	80,000	100,000	249,000	80,000	120,000	126,000	80,000	160,000	173,000	80,000	400,000
		Miscarriage/abortion			100,000			120,000			150,000			400,000

*corrected from 160,000 in the Prakas

Sources: [80]^a^, [81]^b^, [82]^c^

## Footnotes

1 GIZ costs were adjusted using the ratio of cost inputs in this study to projected total costs using GIZ unit cost data.
